# Functional Anatomy, Impact Behavior and Energy Dissipation of the Peel of *Citrus* × *limon*: A Comparison of *Citrus* × *limon* and *Citrus maxima*

**DOI:** 10.3390/plants11070991

**Published:** 2022-04-05

**Authors:** Maximilian Jentzsch, Sarah Becker, Marc Thielen, Thomas Speck

**Affiliations:** 1 Plant Biomechanics Group, Faculty of Biology, Botanic Garden, University of Freiburg, Schänzlestraße 1, D-79104 Freiburg, Germany; sarah@becker-ms.de (S.B.); marc.thielen@biologie.uni-freiburg.de (M.T.); thomas.speck@biologie.uni-freiburg.de (T.S.); 2 Cluster of Excellence livMatS @ FIT, Georges-Köhler-Allee 105, D-79110 Freiburg, Germany; 3 Department of Mechanical Engineering, Westphalian University of Applied Sciences, Münsterstraße 265, D-46397 Bocholt, Germany; 4 Freiburg Materials Research Center (FMF), Stefan-Meier-Straße 21, D-79104 Freiburg, Germany; 5 Freiburg Center for Interactive Materials and Bioinspired Technologies (FIT), Georges-Köhler-Allee 105, D-79110 Freiburg, Germany

**Keywords:** *Citrus* × *limon*, lemon, peel, drop weight test, energy dissipation, damping materials, functionally graded materials (FGM)

## Abstract

This study analyzes the impact behavior of lemon peel (*Citrus* × *limon*) and investigates its functional morphology compared with the anatomy of pomelo peel (*Citrus* *maxima*). Both fruit peels consist mainly of parenchyma structured by a density gradient. In order to characterize the lemon peel, both energy dissipation and transmitted force are determined by conducting drop weight tests at different impact strengths (0.15–0.74 J). Fresh and freeze-dried samples were used to investigate the influence on the mechanics of peel tissue’s water content. The samples of lemon peel dissipate significantly more kinetic energy in the freeze-dried state than in the fresh state. Fresh lemon samples experience a higher impulse than freeze-dried samples at the same momentum. Drop weight tests results show that fresh lemon samples have a significantly longer impact duration and lower transmitted force than freeze-dried samples. With higher impact energy (0.74 J) the impact behavior becomes more plastic, and a greater fraction of the kinetic energy is dissipated. Lemon peel has pronounced energy dissipation properties, even though the peel is relatively thin and lemon fruits are comparably light. The cell arrangement of citrus peel tissue can serve as a model for bio-inspired, functional graded materials in technical foams with high energy dissipation.

## 1. Introduction

In this study we test the energy dissipation properties of the lemon peel (*Citrus* × *limon*) to verify if it has similar properties to the much thicker peel of the pomelo (*Citrus maxima*), which has been shown to be able to dissipate large amounts of kinetic energy resulting from impact events. For comparison of the peels of the two species, we refer to data of pomelo peels from literature [1,2,3] and compare them with experimental results of lemon peel samples. Citrus species are one of the most widely grown crops in the world, with hundreds of cultivated varieties. The genus *Citrus* has three basic taxa, pomelos (*Citrus maxima* Burm.), citrons (*Citrus medica* L.) and mandarins (*Citrus reticulata* Blanco). *Citrus* species and cultivars have been bred through a series of interspecific crosses, resulting in great diversity within the genus *Citrus* [4,5,6,7]. Molecular marker data suggest that the lemon (*Citrus* × *limon* (L.) Burm.fil.) is a crossbreed between sour orange (*Citrus* × *aurantium* L.) and citron (*Citrus medica* L.), with some marker fragments indicating a polyphyletic origin [8,9]. Lemons are often excluded from scientific research, except in genetics and olfactory studies, since they are considered to be “breeding artifacts”. However, because citrus peels serve as a promising model for bio-inspired materials, it would be advantageous to analyze the lemon peel and furthermore compare its impact behavior to that of the pomelo. As such, the aim of this study is to perform a biomechanical and structural comparison of the lemon peel with the peel of pomelo to investigate whether the lemon peel has a comparable structural setup and similar energy dissipation properties as the pomelo peel. For the latter purpose, a series of drop-weight tests with different drop heights is performed and the peel samples are tested in fresh and freeze-dried states.

A more general view of fruits in their natural environment shows that one important function of fruits is to attract vector animals to distribute the seed and spread the plant. The peel protects the fruit from dehydration, mutagenic ultraviolet (UV) radiation and provides mechanical protection. For example, the fruit of a pomelo (*Citrus maxima*), which can weigh up to 6 kg [10], grows on trees of a height of 5–15 m [11]. This means that the peel has to deal with the kinetic energy of impact after being shed. The pomelo fruit can drop from heights of 10 m without any significant outer damage to five out of six fruits [12]. Without energy dissipation, the fruit would rupture on impact; microbes, fungi or bacteria can subsequently infest the pulp [13] and make the fruit less attractive or even inedible for vector animals (e.g., elephants, fruitivorous bats, monkeys, bears, etc.) [14,15]. Therefore, effective impact damping is essential for fruit and seed dispersal.

**Figure 1:**
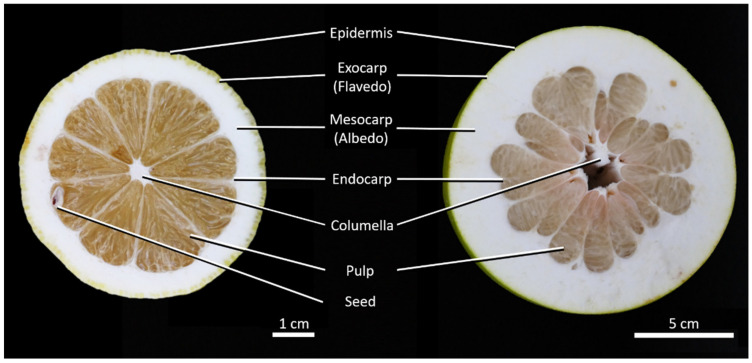
Cross section of *Citrus* × *limon* (**left**) and *Citrus maxima* (**right**).

Citrus fruits consist of pulp, seeds, a central axis, the columella and the peel which can be further subdivided into epidermis, the parenchymatous flavedo (exocarp), albedo (mesocarp) and endocarp (Figure 1). There are also oil glands in the flavedo and vascular bundles running through the entire peel [16,17]. Depending on the species or cultivar, the proportions of the individual tissues of the fruit may differ greatly, and even the fruits as such vary markedly in size and shape.

In addition, the peel of citrus fruits usually has many intercellular spaces in the albedo that influence the properties of the peel structure. Scott and Baker [17] describe that meristematic cells in the young albedo of navel oranges are typically tetrakaidekahedral in form (a 14-faced polyhedron); as they expand, they become spherical, and intercellular spaces appear. The albedo cells do not grow fast enough compared to the whole peel, thus the intercellular spaces expand enormously [17].

Previous studies on *Citrus* × *limon* ‘Eureka’ considered the effects of impact and compression tests of the peel on whole fruits under different storage conditions [18]. The surface damage was assessed visually. The major damage from impact loading occurred in the tissue around the oil glands, in the upper flavedo and in the albedo [18]. The peel of pomelo (*Citrus maxima*) has already been examined in detail: free fall tests with whole fruits and tensile tests with peel samples were carried out [12]. Additionally, drop-weight tests of peel samples [3], compression tests with peel samples have been performed [2] and the peel structure of different varieties of pomelo have been compared [19]. Internal strain mapping (X-ray tomographic imaging and digital volume correlation) has been made via in-situ stepwise uniaxial compression tests of peel samples [20], and the viscoelastic behavior of the peel has been characterized by mathematical models [1]. First replicas of the peel structure on silica have been made [21], and pomelo-inspired metallic foams [12] and composites have been designed [22,23]. Moreover, there are initial additively manufactured porous polymer cubes inspired by the pomelo peel [19].

Since the peel in particular is responsible for protecting the fruit from mechanical stress, a model for bio-inspired technical damping materials can be created by taking inspiration from the structural set-up of the peel. By this, the peel structure of citrus fruits can serve as a model for impact-absorbing safety components, e.g., for transportation systems [22], in the field of packing technology [22] or for personal protection gear such as helmets or knee pads [12,22,24]. For reasons of material savings, it is for a technical transfer of great interest to analyze if the significantly thinner peel of the lemon can perform comparably good as the thicker peel of the pomelo.

## 2. Results

### 2.1. Anatomy

**Figure 2:**
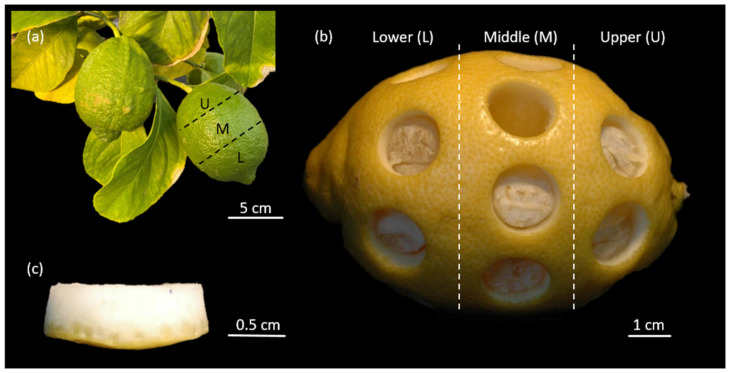
Lemon (*Citrus* × *limon*) fruit divided into three parts (lower (L), middle (M) and upper part (U)), (**a**) showing unripe lemon fruits in their natural orientation on the tree. (**b**) Ripe *Citrus* × *limon* with samples cut out using a cork borer. (**c**) Freshly cut-out sample of the peel of *Citrus* × *limon*.

The purchased lemon fruits had an average length of 91.06 ± 5.89 mm and an average mass of 166 ± 10 g. The fresh lemon peel had an average relative water content (RWC) of 65.95 ± 6.45%. The lower part of the lemon fruit is more conical shaped and has a greater curvature than the other two parts of the fruit; this is related to the prolate shape of the whole fruit (cf. Figure 2b).

**Figure 3:**
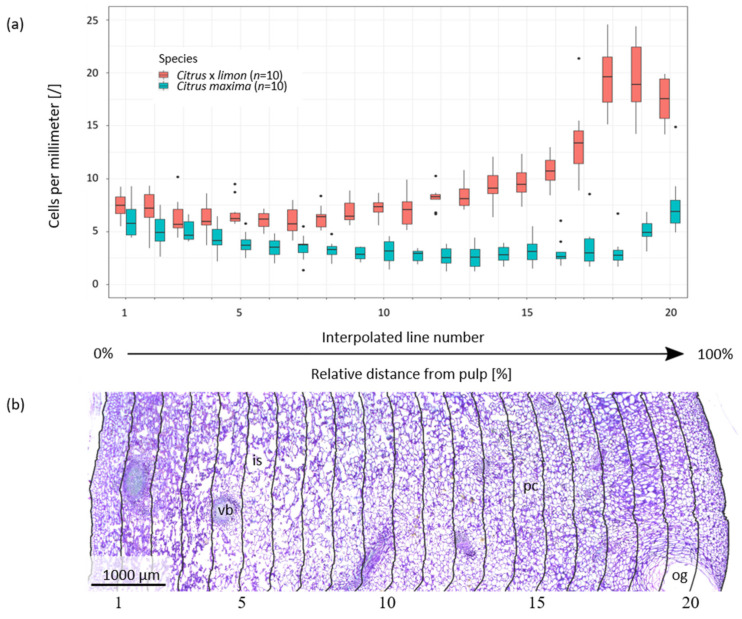
(**a**) Average cell number per millimeter counted at interpolated lines in thin sections of *Citrus* × *limon* and *Citrus maxima*. (**b**) Cross thin section of the peel *Citrus* × *limon* stained with toluidine blue, showing the 20 interpolated lines along which the cell numbers have been counted. The cross thin section of *Citrus* × *limon* is characteristic for both species from a qualitative point of view. The peel of both species mainly consists of oil glands (og), parenchyma (pc), intercellular spaces (is) and vascular bundles (vb). The cell density shows a gradual decrease from the outside of the peel (right) to the inside. In the flavedo close to the epidermis (right) the cells are visibly more densely arranged than in the part of the albedo which is close to the endocarp (left).

To understand the differences in impact behavior of *Citrus* × *limon* and *Citrus maxima*, it is not sufficient to only compare the mechanical properties, rather it is necessary to also analyze and compare the underlying peel structure representing the structural basis for mechanical behavior. Within the structural basis, if the relative proportion of the peel in comparison to the pulp and the central axis is considered, it is noticeable that the median of the peel proportion of *Citrus* × *limon* with 36.42% (interquartile range (IQR):4.82%) is significantly smaller (*p* < 0.001) than that of *Citrus maxima* with 46.93% (IQR:13.66%). Moreover, the absolute peel thickness of *Citrus* × *limon* with 5.26 mm (IQR:1.41 mm) differs significantly (*p* < 0.001) from the peel thickness of *Citrus maxima* (12.74 mm (IQR:6.01 mm)) (peel thickness of *Citrus maxima* is calculated by the data of [1,3]). The average peel density of *Citrus* × *limon* is almost twice as high as *Citrus maxima*, with 818.36 ± 41.54 kg/m^3^ compared to 417.72 ± 60.02 kg/m^3^, respectively. Thin cross sections were made to analyze the radial cell arrangement from the pulp to the epidermis. Figure 3 shows a comparison of the number of cells per millimeter, in relation to the distance from the pulp of *Citrus* × *limon* and *Citrus maxima*. The number of cells per millimeter for *Citrus* × *limon* increases strongly with increasing distance from the pulp, after a short (initial) decrease (Figure 3a). Comparing the number of cells per millimeter of *Citrus* × *limon* with those of *Citrus maxima*, the average number of cells of *Citrus maxima* is lower. The difference between the average cell numbers per millimeter in *Citrus* × *limon* and *Citrus maxima* is especially large in the areas close to the epidermis (flavedo); however, close to the pulp in the endocarp region (interpolated lines 1–3), only the number of cells per millimeter at the second interpolated line shows a significant difference between *Citrus* × *limon* and *Citrus maxima* (0.01 ≤ *p* < 0.05). In the flavedo region, which is represented in the median by the last four (IQR:0) interpolated lines (interpolated lines 17–20) in *Citrus* × *limon* and by the last (IQR:1) interpolated line in *Citrus maxima*, the cells are more densely packed than in the central peel region farther away from the epidermis, i.e., the albedo region, where more intercellular spaces are visible. The toluidine blue-stained thin section of the peel of *Citrus* × *limon* (Figure 3b) illustrates the gradual change in cell arrangement from the endocarp (left) to the epidermis (right), which from a qualitative perspective does not differ from that of *Citrus maxima* [2], except for absolute sample thickness and tissue density. The cross section shows that the peels of both fruits mainly consist of parenchyma cells (pc), vascular bundles (vb), oil glands (og) and intercellular spaces (is) (Figure 3b).

### 2.2. Sample Thickness

**Figure 4:**
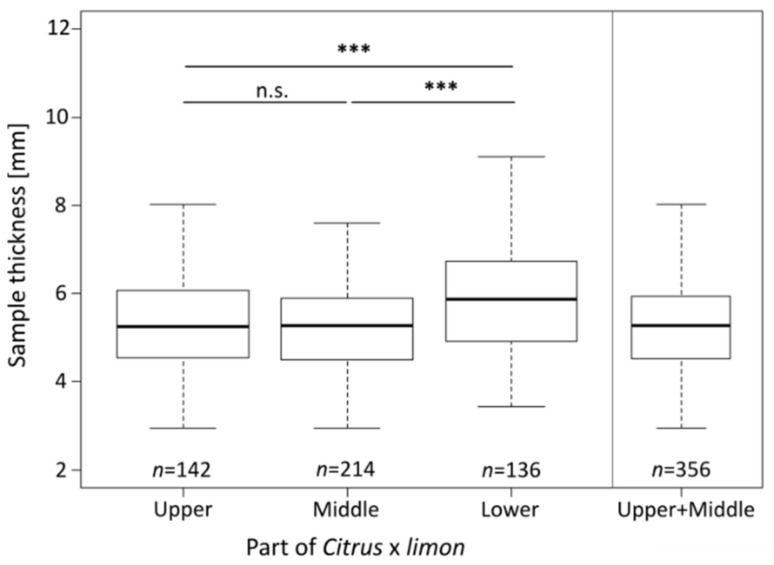
Sample thickness according to the parts within the fruit. The sample thickness of each sample was measured three times with a digital caliper. The level of statistical significance is indicated in the figure as follows: n.s.: *p* ≥ 0.05; ***: *p* < 0.001.

Cylindrical samples were taken from the three regions of the fruit (upper, middle and lower parts; see also Figure 2b). Prior to any mechanical testing, the sample thickness was measured; the results show that the samples from the lower part, with a thickness of 5.87 mm (IQR:1.80 mm), are significantly thicker than the samples from the other two parts (upper: 5.25 mm (IQR:1.53 mm), middle: 5.26 mm (IQR:1.37 mm)) (*p* < 0.001) (Figure 4). There was no significant difference in the thickness of the samples between the upper and middle parts. Therefore, for further analysis the samples from the upper and middle parts, which have a thickness of 5.26 mm (IQR:1.41 mm), were pooled. This significant difference between samples from the lower part and the other two parts is probably due to the higher curvature of the fruit in the lower part, which can be observed in Figure 2b. Therefore, the samples of the lower part were rejected from further investigations.

### 2.3. Drop-Weight Tests

**Figure 5:**
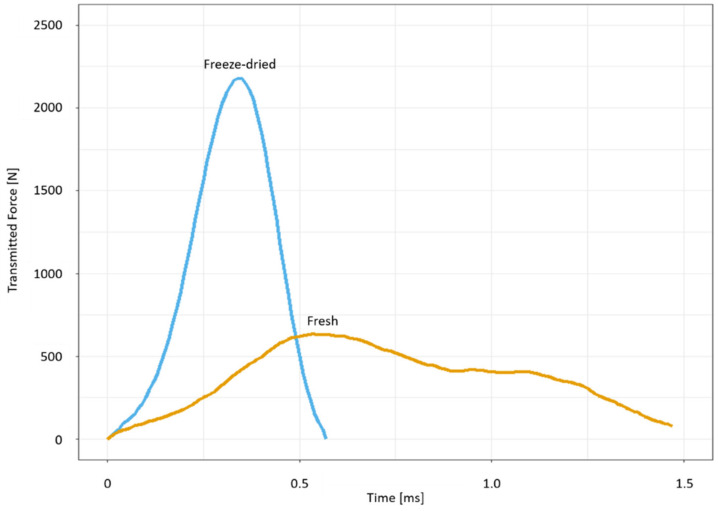
Characteristic force-time diagrams of a drop-weight test for a fresh and a freeze-dried sample of *Citrus* × *limon*. The area under each graph characterizes the impulse of the impact. The impactor (mass = 0.061 kg) was dropped from a height of 0.81 m onto the samples.

For the drop-weight tests, fresh and freeze-dried samples were used. During the freeze-drying process the samples showed a lateral shrinkage in comparison to the fresh-state lemon sample, with a resulting median value of 3.39% (IQR:8.10%) shrinkage. The comparison of the force-time curves of the impact on fresh and freeze-dried samples shows differences in the shapes of the curves (Figure 5). For the fresh samples, the force increases until it reaches its maximum after around 0.5 ms, then the curve slowly flattens. For freeze-dried samples, the force rises more quickly and reaches its maximum after around 0.3 ms; afterwards, the force decreases much faster than it does with the fresh samples. The difference of the two sample preparations is reflected in the mean impact duration, which is 1.31 ± 0.09 ms for fresh and 0.97 ± 0.30 ms for freeze-dried samples. Thus, the average impact duration for fresh samples typically lasts 35% longer than that of the freeze-dried samples.

**Figure 6:**
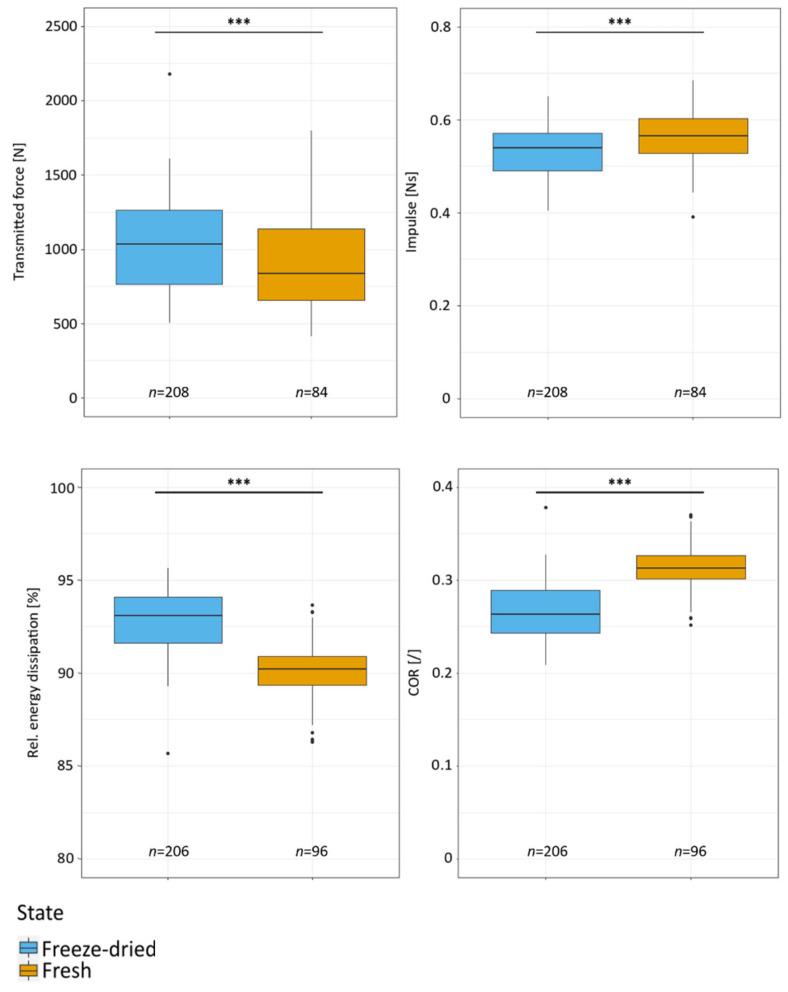
Comparison of fresh and freeze-dried samples of *Citrus* × *limon*. Drop-weight tests with an impactor (mass 0.061 kg) dropped from a 0.81-m height. The level of statistical significance is indicated in the figure as follows: n.s.: *p* ≥ 0.05; ***: *p* < 0.001.

The transmitted force maximum of the impact has a median value of 837.13 N (IQR:480.12 N) for fresh samples (Figure 6). For freeze-dried samples the force maximum is significantly higher with 1033.08 N (IQR:498.98 N). The interquartile range of the median forces is high for both fresh samples (57.35%) and freeze-dried samples (48.30%).

A comparison of the impulses of the impact shows that the fresh samples experienced significantly larger (*p* < 0.001) impulses (0.56 ± 0.05 Ns) than the freeze-dried samples (0.53 ± 0.06 Ns) (Figure 6). This is also reflected in Figure 5 where the impulse can be seen as an integral of the curve of the force-time diagram. The relative dissipated energy in the freeze-dried samples, with a median of 93.09% (IQR:2.47%), is significantly higher (*p* < 0.001) than in the fresh samples with an average of 90.21% (IQR:1.55%). In order to describe the impact behavior and how much kinetic energy returns to the impactor, the coefficient of restitution (COR), which describes the elasticity of an impact, is determined in the drop-weight tests (for a definition of COR and the other mechanical parameters we refer to Section 4.4). When considering the COR, it becomes clear that the fresh samples have a significantly higher median value than the freeze-dried samples. The fresh samples show a median COR value of 0.31 (IQR:0.02) compared to the freeze-dried samples’ COR value of 0.26 (IQR:0.05). Additionally, the fresh samples show a permanent deformation of 3.15% (IQR:4.43%), measured 3 min after the impact, whereas the freeze-dried samples have a permanent deformation of 8.33% (IQR:7.27%).

### 2.4. Different Drop Heights

**Figure 7:**
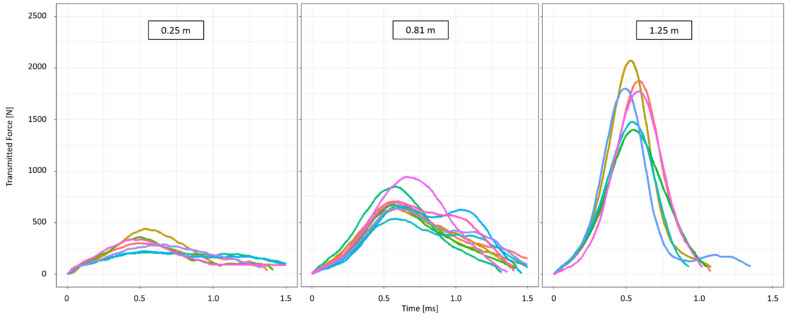
Force-time diagrams for fresh *Citrus* × *limon* samples at different drop heights (0.25 m (*n* = 7), 0.81 m (*n* = 10), 1.25 m (*n* = 6)). For clarity, only 10 out of 208 samples tested at a drop height of 0.81 m are plotted. The drop-weight tests were performed with a cylindrical impactor (mass = 0.61 kg).

In order to investigate how fresh and freeze-dried peel samples of *Citrus* × *limon* behave under different impact conditions, drop-weight tests with different drop heights (0.25 m–1.25 m) were carried out. These varying height tests resulted in a kinetic impact energy between 0.15–0.74 J. The force-time curves of the samples dropped from the lowest height showed an impact duration of 1.48 ± 0.32 ms. At the drop height of 0.81 m, the force-time curves from the samples were similar in terms of impact duration (1.31 ± 0.09 ms) (Figure 7). The median force peak with 298.28 N (IQR:89.44 N) (0.25 m drop height) is significantly lower than the force peak of the 0.81-m drop height (837.13 N (IQR:480.12 N)); double peaks, or at least plateaus, occur more frequently at a drop height of 0.81 m. When comparing the force-time curve of the highest drop height (1.25 m) with the force-time curve of the drop height at 0.81 m, it becomes clear that the maximum force peak for the highest drop height is significantly larger (1785.86 N (IQR:309.24 N)). This means that the force increase here is faster, and the impact as a whole is shorter (1.20 ± 0.32 ms) than the impact with a drop height of 0.81 m. The impact duration is also shorter than that of the lowest drop height (0.25 m). It should be added, however, that even at a drop height of 1.25 m, plateaus are observed occasionally.

**Figure 8:**
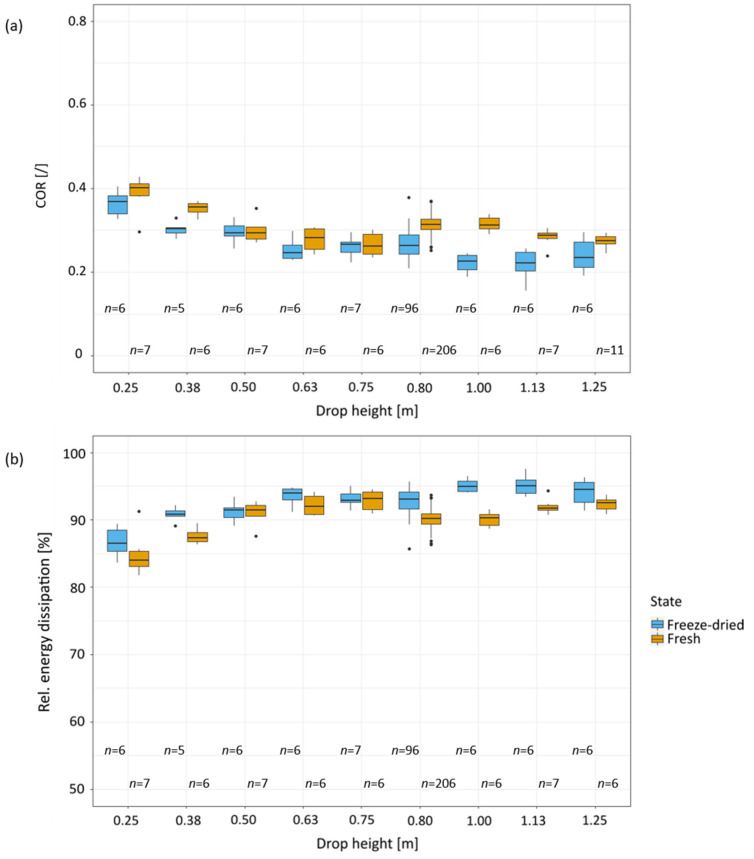
(**a**) Coefficient of restitution (COR) of the impact on fresh and freeze-dried samples of *Citrus* × *limon* for different drop heights, and (**b**) relative energy dissipation of fresh and freeze-dried samples of *Citrus* × *limon* for different drop heights. All drop-weight tests were performed with a cylindrical impactor with a mass of 0.061 kg.

Figure 8a shows that with an increasing drop height the COR values of the fresh samples tend to decrease. There are two exceptions at the drop height of 0.80 m and 1 m at which the COR values increase. For the drop heights of 1.13 m and 1.25 m the COR values decrease again with increasing drop height, in comparison to the COR values before. The COR values at the drop heights of 1.13 m and 1.25 m are similar to the COR values at drop heights between 0.5 m and 0.75 m. The highest median COR value is found at a drop height of 0.25 m (0.40 (IQR:0.03)), and the lowest COR value occurs at a drop height of 0.75 m (0.26 (IQR:0.05)). The COR values of the freeze-dried samples also decrease with increasing drop heights. The COR values of the freeze-dried samples are lower than the values of fresh samples. However, no significant difference can be detected between the median COR values when comparing one drop height to the preceding or following drop height. The largest median COR value was determined for the lowest height (0.25 m) (0.37 (IQR:0.04)). The lowest median COR value in freeze-dried samples was recorded at a height of 1.13 m (0.22 (IQR:0.04)).

With increasing drop height, the relative energy dissipation of the samples increases (Figure 8b). The median of the energy dissipation for different drop heights behaves oppositely to the COR values, which is due to the fact that the energy dissipation can be calculated as 1 minus the squared COR value. The largest energy dissipation of fresh samples is measured at a drop height of 0.75 m and amounts to 93.14% (IQR:2.53%). The median maximum energy dissipation for freeze-dried samples is measured with 95.06% (IQR:1.99%) at a drop height of 1.13 m.

## 3. Discussion

In the present study, we analyzed the impact behavior of the peel of *Citrus* × *limon* to perform a biomechanical comparison between the relatively thin peel of *Citrus* × *limon* and the much thicker peel of *Citrus maxima*. Our research of *Citrus* × *limon* shows that the peel of the lower fruit part (L) (lower: 5.87 mm (IQR:1.80 mm)) is thicker than in the middle (M) and upper parts (U), (upper: 5.25 mm (IQR:1.53 mm), middle: 5.26 mm (IQR:1.37 mm)) (Figure 2 and Figure 4). This may be related to the prolate shape of the fruit (Section 4.3). For nearly spherical *Citrus maxima* fruits Thielen et al. [1] could not find any significant difference for peel thickness in the three tested parts. The anatomical comparison of the peels of *Citrus* × *limon* and *Citrus maxima* demonstrates that the peel of *Citrus* × *limon* has a significantly thinner peel, which is on average only half as thick as the peel of *Citrus maxima* (Section 2.1). Morever, *Citrus* × *limon* has a significantly lower relative contribution of the peel to the fruit’s volume (Section 2.1). The peel thickness for fresh samples of *Citrus maxima* is calculated from supplementary data provided in Thielen et al. [3]. The peel density in *Citrus* × *limon* is almost twice as high as that in *Citrus maxima* (Section 2.1). In both species, the number of cells per millimeter expands with increasing distance from the pulp, causing a gradient in peel density (Figure 3). For *Citrus* × *limon* there exists a significant increase in the amount of cells, after an initially significant decrease, in comparison to the amount of cells close to the pulp (Section 2.1). In contrast to *Citrus × limon*, *Citrus maxima* shows after an initially significant decrease in the amount of cells a relatively constant cell distribution. Furthermore, the cell density begins to increase in the flavedo again (Figure 3). Yang et al. [19] describe a similar decrease in the cell distribution of different varieties of *Citrus maxima*. They also observed an increase in the cell density towards the epidermis, but they divided the peel into coarser steps (20% steps) and did not describe the increase in higher spatial resolution and detail. In *Citrus* × *limon* the number of cells per millimeter shows a steeper increase, especially in the last third of the peel (Figure 3) towards the epidermis, than that in *Citrus maxima*. Additionally, *Citrus maxima*’s entire peel contains a much higher amount of air-filled intercellular spaces [2]. In comparison of the absolute peel thicknesses, the flavedo of both fruits have approximately the same thickness of 1 mm (Section 2.1). As a result of the different peel thicknesses, however, the peel of *Citrus maxima* consists of approximately 5% flavedo, whereas *Citrus × limon*´s flavedo contributes nearly 20% to the total peel thickness (Section 2.1). From an engineering perspective, the peel structure can be described as a sandwich structure. Both citrus fruit peels have an approximately 1-millimetre-thick outer covering layer (flavedo), a core layer (albedo) of varying, gradually changing density and thickness, and at the transition to the pulp, a further thin covering layer (endocarp) (Section 2.1). Additionally, both peels are reinforced by a 3D network of strengthening bundles running through the structure (vascular bundles). The cell density gradient determined in our results resembles a cellular foam core structure [25] in a functionally graded material (FGM) [26], which allows the peel to dissipate energy through plastic deformation. This biological sandwich structure envelops and protects the pulp and seeds from potentially damaging environmental influences. It also protects the fruit from mechanical stresses such as impacts [27]. The geometry of the single cells is less important than the ratio of relative density and solid density for the mechanical properties [25,28,29].

The similarities and differences found in the flavedo structure suggest that the biomechanical differences between *Citrus* × *limon* and *Citrus maxima* peel are a result of the structural composition of the albedo (cellular foam core structure) (Section 2.1). The thickness of the albedo in *Citrus* × *limon* is both relatively and absolutely significantly lower than the thickness of the albedo in *Citrus maxima* (Section 2.1). The more graded transition from albedo to flavedo in *Citrus* × *limon* is responsible for its functional morphological difference from *Citrus maxima*, and mitigates impact damage [30,31,32]. This graded albedo contributes to the high energy dissipation in the relatively thin *Citrus* × *limon* peel (Figure 6 and Figure 8). *Citrus maxima* compensates for the less graded transition in the peel with its thicker peel and its less densely arranged albedo. The peculiarities of the peel structure are also the reason why *Citrus maxima* achieves a very high energy dissipation [19]. The difference of *Citrus* × *limon* is probably due to the fact that the fruit is derived from a species cross, and it has been bred intensively [5,8]. *Citrus maxima*, on the other hand, represents one of three basic taxa with relatively little breeding influence [5]. Based on this intensive breeding, it can be hypothesized that the reported differences in peel thickness and anatomy of *Citrus* × *limon* are a consequence of species crossing and selection breeding. Selection breeding in general favors the part of the fruit for the interest of breeders (i.e., nutrients or pulp), and decreases the contribution of the other fruit tissues (i.e., seeds, peel and columella) [33,34,35].

Mechanical tests were performed on fresh and freeze-dried samples of *Citrus* × *limon* peel (Section 4.4) and compared to values of *Citrus maxima* by Thielen et al. [3]. The lower lateral shrinkage in freeze-dried samples of the denser *Citrus* × *limon* (Section 2.2) can be attributed to a slightly different freeze-drying method used (Section 4.4). This method is used to avoid freeze-cracking. In the peel parenchyma cells, the influence of the water content mainly depends on cell wall properties and turgor pressure. Freeze-drying results in dehydration of the entire cell (and cell wall) by sublimating the frozen water from the vacuole, the protoplast and the cell wall matrix [3,24,36,37,38]. The pressure loss of the individual cells and the contraction stresses are results of the water loss in the tissue. This also explains the observed shrinkage of the peel samples [3].

The comparison of mechanical properties between fresh and freeze-dried samples of *Citrus* × *limon* (Section 2.3) shows that significantly less force is transmitted through fresh peel samples. We also found out that the impulse is significantly larger than that in the freeze-dried samples (Figure 6). The freeze-dried samples dissipate significantly more energy than fresh samples (93.09% (IQR:2.47%) compared to 90.21% (IQR:1.55%)) (Figure 6). They also show a more plastic impact behavior (COR: 0.26 (IQR:0.05)) than the fresh samples (COR: 0.31 (IQR:0.02)) (Figure 6). The higher transmitted force of freeze-dried samples (Figure 6) is caused by freeze-drying, which removes the water from the tissue. Due to the lack of water in the cell walls, the cellulose microfibrils form more H-bonds and can no longer slide past each other. The result is a stiffening of cell walls, cells and finally the tissue comprised of these cells [36,37]. The stiffer structure results in a more plastic impact in which more kinetic energy is dissipated by plastic deformation (Section 2.3).

There are two ways to generate an equally high impulse: (1) by a large force acting over a short period of time, or (2) by a small force acting over a longer period of time [24]. For a given impulse, it is biologically advantageous for the fruit peel if the duration of the impact is prolonged. The prolonged impact keeps the forces low and subcritical, thus avoiding damage. The impact duration depends on the thickness and stiffness of the peel samples. Fresh samples (Figure 5) showed a significantly longer impact duration, since the peel is thicker and less stiff than the peel of freeze-dried samples. Additionally, more reaction time is needed to change the momentum and dissipate the energy. Viscoelastic effects in the fresh samples also support a more elastic impact, although the viscoelastic effects decrease with increasing drop height as a result of the higher impact velocity, as more cell bursting occurs [1].

The comparison of the force-time curves of the fresh samples for the minimum and maximum drop heights (0.25 m, 1.25 m) shows that with increasing drop height, the transmitted force increases almost by a factor of six. The impact duration also decreases by about 20% (Figure 7). The decrease in impact duration and the higher transmitted force may suggest that the samples collapse with increasing drop height. However, this is not probable since the energy dissipation and the COR values increase with increasing drop height until they reach a plateau (Figure 8). Both parameters, COR and energy dissipation, do not increase significantly with the previous and subsequent drop heights, hence an abrupt collapse of the specimens cannot be assumed (Section 2.4).

Comparing the impact duration of *Citrus* × *limon* (Section 2.4) with *Citrus maxima* [3], the impact duration of *Citrus* × *limon* is apparently smaller. The lower impact duration can be explained by the lower peel thickness [24,39]. However, while the impact duration of *Citrus* × *limon* is shortened by about 20% for fresh samples in comparison with the minimum and maximum drop heights (0.25 m, 1.25 m) (Section 2.4), the impact duration is shortened by a similar (and partly higher) amount for *Citrus maxima* [3]. It must also be mentioned that the median transmitted force for fresh samples at a drop height of 1.25 m is 2–3 times greater for *Citrus* × *limon* (Section 2.4) than it is for *Citrus maxima* [3].

**Figure 9:**
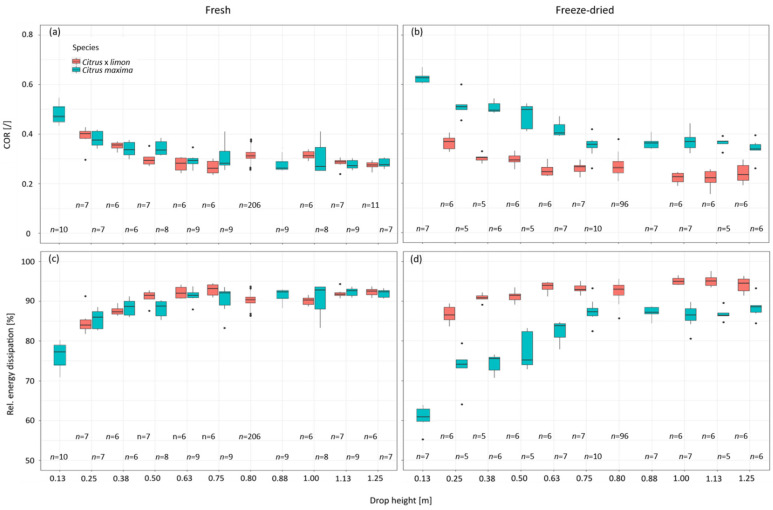
(**a**,**b**) Coefficient of restitution (COR) of fresh and freeze-dried samples, and relative dissipated energy of the impact (**c**,**d**) on *Citrus* × *limon* and *Citrus maxima* for different drop heights. All drop-weight tests were carried out with a cylindrical impactor having a mass of 0.061 kg. The data of *Citrus maxima* are taken from [3].

While dehydration of the cell wall and the loss of turgor in *Citrus maxima* result in a more elastic impact and higher energy dissipation [3], the peel of *Citrus* × *limon* demonstrates an inverse behavior (Figure 9). A comparison between the COR values of fresh *Citrus* × *limon* peel samples and of fresh *Citrus maxima* peel samples show very similar values for all of the tested drop heights (Figure 9a,c). In the freeze-dried state, peel samples of *Citrus* × *limon* have a significantly lower COR for all tested drop heights compared to *Citrus maxima*. The peel samples of *Citrus* × *limon* behave in a more plastic manner, and result in significantly higher relative energy dissipation compared to peel samples of *Citrus maxima* (Figure 9b,d). Freeze-drying of the peel of *Citrus maxima* results in a weakening of the structure, and is caused by the significantly lower density and larger intercellular space. In contrast, freeze-drying strengthens the peel structure of *Citrus* × *limon*. The more graded transition from albedo to flavedo and the greater cell density with a relatively thicker proportion of dense flavedo in *Citrus* × *limon* (Section 2.1) provide a better stress distribution throughout the sample in the freeze-dried state compared to the constant, less densely arranged albedo in *Citrus maxima*. The denser peel with smoother graded albedo of *Citrus* × *limon* becomes stiffer with freeze-drying (Section 2.3). Since there are more cells due to the smaller intercellular space, the peel of *Citrus* × *limon* remains more stable in the freeze-dried state than the peel of *Citrus maxima* (Section 2.1). Furthermore, the impact in *Citrus* × *limon* becomes more plastic as a result of the more rigid structure, so that more energy is dissipated than in its fresh state (Figure 9a,b). The more plastic impact behavior of the freeze-dried lemon samples is also shown by the finding that the fresh samples experience a permanent deformation about 2.5 times smaller than freeze-dried samples at the same impact height. After an impact from a drop height of 0.81 m, fresh samples have a permanent deformation of 3.15% (IQR:4.43%). In comparison, freeze-dried samples showed a permanent deformation of 8.33% (IQR:7.27%) (Section 2.3).

The peel structure of both fruits is relatively similar, and it can serve as inspiration for the design of technical sandwich structures (Section 2.1). While the smoother gradation of *Citrus* × *limon* is the decisive factor, the peel of *Citrus maxima* is characterized by a thicker but less dense albedo layer which is less graded (Figure 9). Nevertheless, the thicker and less dense albedo still enables the peel of *Citrus maxima* to generate impulses with a longer impact duration at a lower transmitted force [3]. The best solution for a transfer to bio-inspired technical damping materials systems is by using a combination of the properties of both fruit peels. Thus, a thicker peel with a stronger gradient of the cells from albedo to flavedo would be most desirable and would provide optimal impact protection [30,40]. Depending on the application, different optimizations are necessary [40]. If thickness is a limiting restriction, a smooth transition of the albedo, such as in the peel of *Citrus* × *limon,* is a very promising model. It is important to ensure that the transmitted force remains subcritical. Such applications could be, for example, an athlete’s mouthguard [39,41] or in packing technology [22].

## 4. Materials and Methods

### 4.1. Plant Material

Ripe Spanish lemons (*Citrus* × *limon* ‘primofiore’) were purchased from a local supplier (Frische Brüder Germany GmbH, Freiburg, Germany) and stored at ambient conditions until examination (1–5 days). Chinese pomelos (*Citrus maxima* ‘honey pomelo’) were purchased from a local supermarket. Only fruits without any visible damage or fungal infections were used in the different test series, in fresh and freeze-dried states, and dropped from different drop heights (0.13–1.25 m).

### 4.2. Anatomy

In order to compare the mechanical properties of *Citrus* × *limon* with those of *Citrus maxima*, the peel structure of the two fruits has to first be compared. The lengths and weights of the whole fruits were measured. Additionally, the relative water content (RWC) of the peel was determined according to [42]. In 70 cylindrical peel samples from randomly chosen parts, the fresh and dried weights of the samples were measured and normated, and the average relative water content of the fresh samples were determined. Twelve cross sectional images of the fruit of *Citrus* × *limon* and 10 of *Citrus maxima* were made (Figure 1). Then, the proportions of peel, pulp and columella were evaluated using ImageJ (version 1.52a). In addition, 17 cubic samples of 10 fruits were taken from each of the two species, and the peel density in the fresh state was determined by measuring mass and volume of the peel samples. In order to determine the radial cell density of the peels, 10 radial peel samples of each taxon were fixated in formalin-acetic acid alcohol (FAA) for three days. In a subsequent ethanol series, the samples were dehydrated (70% ethanol for 24 h, 90% ethanol for 2 h, 100% ethanol for 1.5 h). After dehydration, samples were embedded in Technovit 7100 (Heraeus Kulzer GmbH, Hanau, Germany). The polymerized samples were cut into 3–5-micrometre-thick thin sections using a rotary microtome (Leica Mikrosysteme Vertrieb GmbH, Wetzlar, Germany). For determining the radial cell density, the thin sections were stained with toluidine blue and images of the sections were taken. Using the software Inkscape (version 0.92.4), the outer edges (epidermis and endocarp) were marked with a line on each (epidermis and endocarp), and 20 interpolated lines were inserted at equal distances between epidermis and endocarp [1]. Since each peel sample had a different thickness (distance from endocarp to epidermis), relative distances were chosen for the interpolation lines to compare different sections. The cells cut by these interpolation lines were counted. The number of cells was divided by the length of the interpolation line minus the length of “disturbing factors” such as vascular bundles or oil glands.

### 4.3. Sample Preparation

The 640 samples (lower part *n* = 136, middle part *n* = 293, upper part *n* = 211) for the impact tests (fresh and freeze-dried states) were taken from 32 lemons. The fruits were divided into three equal parts: lower (L), middle (M) and upper parts (U), corresponding to the orientation of the unripe fruits on the tree (note, however, that the lower part corresponds to the apical part in botanical terms) (Figure 2a). Between 6 and 12 cylindrical samples were cut out of a fruit with a cork borer (inner diameter 15.31 ± 0.03 mm) (Figure 2b) for each part, and the pulp was removed by a razor blade (Figure 2c). After preparation, the fresh samples were tested within 10 min to prevent dessication (lower part *n* = 136, middle part *n* = 214, upper part *n* = 142). The sample thickness was measured three times and averaged for each sample before testing. It was measured three minutes after the impact using a digital caliper. In order to obtain information on the energy dissipation capacity of the peel, different impact strengths were tested in a test series by dropping the impactor from different heights (0.25–1.25 m).

Before being tested, the remaining 148 samples were cooled down to −27 °C within one day and dried for five days in a freeze dryer at −53 °C, and at a pressure of 3 mbar (Christ Alpha 1–4 LO, Martin Christ Gefriertrocknungsanlagen GmbH, Osterode am Harz, Germany). The samples were placed in an airtight storage container and were tested within 36 h (middle part *n* = 79, upper part *n* = 69).

### 4.4. Mechanical Testing

**Figure 10:**
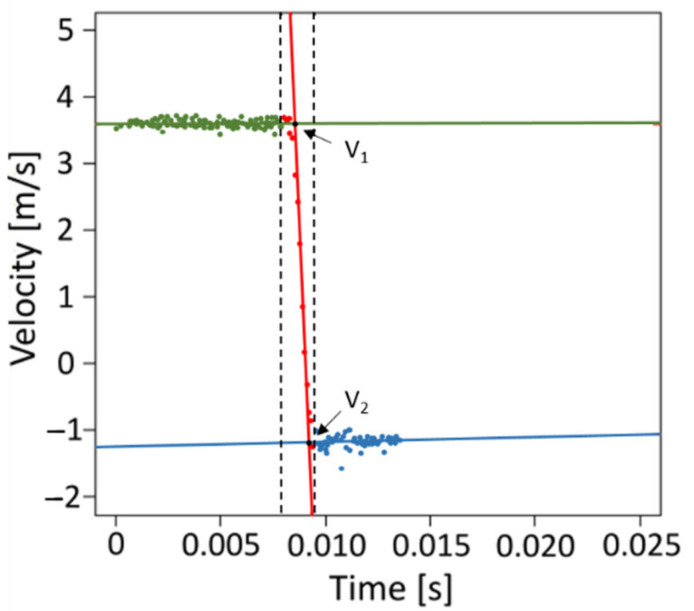
Average velocity of an impactor with mass = 0.061 kg dropped from a height of 0.81 m onto a fresh lemon sample. The impactor’s position is calculated for each point in time by averaging five tracking points on the impactor. The intersections of the regression lines show the velocities immediately before (v_1_) (intersection of the green and red regression lines) and after (v_2_) the impact (red and blue regression lines).

The dynamic tests were performed using a drop-weight tower as described in detail in [3]. With the drop-weight tests, the behavior of the peel under impact loading was analyzed and information about its energy dissipation capacity were obtained. A cylindrical, flat-ended aluminum drop weight was used as an impactor (0.061 kg) and dropped from a defined height onto a peel sample. If no other drop height is indicated, the drop height was 0.81 m. Even though several rebounds of the impactor were recorded upon each impact, only the respective initial impacts were evaluated. The beginning and the end of the impact was determined manually for each drop-weight test from the force-time curves. The force with which the impactor acted on the sample was measured with a frequency of 10 kHz by a 10-kiloNewton force sensor (model 8402, Burster Präzisionsmesstechnik GmbH & Co KG, Gernsbach, Germany) which was placed under the sample and under a steel anvil, as shown in [3]. The measured forces were corrected for the baseline offset calculated from data points before the impact of each drop test. The impact was recorded by high-speed camera (MotionProY4L Mono, Image Solution GmbH, Eningen unter Achalm, Germany) at a frame rate of 10,000 fps. A dot pattern was applied to the impactor to allow video tracking of the impactor’s position. Five random dots of the pattern were selected as tracking points and while recording the impact, the changes of their positions in time were also recorded so that the speed of the impactor could be calculated. The impactor position could be calculated for the complete impact duration by the five tracking points (Figure 10). The position of each measuring point was determined using the software MotionStudio (version 2.11.00, Integrated Design Tools, Inc., Tallahassee, FL, USA). The drop-weight test could be clearly divided into three phases using the impactor’s velocity: before impact, during impact and after impact. The intersection points of the three linear regression lines correspond to the velocity of the impactor immediately before (v_1_) (intersection of green and red regression lines) and after (v_2_) the impact (intersection of red and blue regression lines). A linear regression was assumed since the observed period of time was very short [3].

The absolute value of the ratio of the velocity after the impact (*v*_2_) and the velocity immediately before the impact (*v*_1_) is the coefficient of restitution (*COR*).

(1) $$COR=|\frac{v_{2}}{v_{1}}|$$

The restitution coefficient describes the elasticity of the impact, and can take values between 0 and 1. A COR value of 0 means an ideal *plastic* impact, which means that the peel dissipates all kinetic energy. A COR value of 1, on the other hand, means an ideal *elastic* impact, which means that all the kinetic energy returns to the impactor.

The kinetic energy (*E_kin_*) is calculated as one-half the product of an object’s mass and the square of its velocity.

(2) $$E_{kin}=\frac{1}{2}mv^{2}$$

The relative (rel.) dissipated kinetic energy (*E_diss_*) is the relative ratio of the kinetic energy before (*E_kin_*_1_) and after the impact (*E_kin_*_2_). The rel. energy dissipation is the energy of an impact that becomes dissipated by the impacted object.

(3) $$Rel.\;E_{diss}=\frac{E_{kin1}-E_{kin2}}{E_{kin1}}*100$$

When an impactor hits a non-moving object, the momentum of the impactor is transferred to the object, according to the law of conservation of momentum. The momentum ($\vec{p}$) is defined as the product of mass (*m*) and velocity ($\vec{v}$) of the moving object.

(4) $$\vec{p}=m\vec{v}$$

The impulse (*J*) is the change of momentum over a period of time. The impulse is determined as the integral of the force (*F*) over the impact time, and is measured as the area under the force-time curve during the impact.

(5) $$J=\overset{t_{2}}{\underset{t_{1}}{\int }}F\;dt$$

### 4.5. Statistics

The generated data sets were statistically evaluated using the free software GNU R version 3.6.1 [43]. By using a Shapiro test, the connected data sets were tested for normal distribution. In a subsequent Levene test, the homogeneity of the variances was checked. Afterwards, depending on the size of the group, and whether the data were parametric or non-parametric, various statistical tests were performed. Normally distributed data sets consisting of two or fewer groups were tested for significance of the average values using a T-test, whereas normally distributed data sets with more than two groups were tested for significance of the variances using an ANOVA. If the ANOVA showed a significant difference between the groups, a Tukey HSD test was used to determine which groups had a significant difference. If one or more data sets were not normally distributed, a Wilcoxon Rank Sum test (also called Mann–Whitney U-test) was used for homogeneity of the distributions for groups smaller than or equal to two. A Kruskal–Wallis test was used for significance of the variances for larger groups. If a Kruskals–Wallis test showed significance, a pairwise Wilcoxon Sign-Rank test with Bonferroni correction of the *p*-values was used to check between which groups there was a significant difference. The level of statistical significance is indicated in the figures as follows: n.s.: *p* ≥ 0.05; *: 0.01 ≤ *p* < 0.05; **: 0.001 ≤ *p* < 0.01; ***: *p* < 0.001.

## 5. Conclusions

The results of this study show that the peel of *Citrus* × *limon* has very high energy dissipation capabilities, which are similar to the ones previously reported for the peel of *Citrus maxima* [3,19], although the gradation of the albedo, the sample thickness, the transmitted force and impact duration differ significantly (Section 2). The structures of both fruits can be described as sandwich structures, with a graded foam core in technical terms. While the smoother graded transition is decisive for *Citrus* × *limon*, the peel of *Citrus maxima* shows a thicker but less dense albedo which is less graded (Section 2.1). In further studies, it would be interesting to investigate the importance of the vascular bundles in the peel of *Citrus* × *limon*, in order to determine if they also mechanically support the tissue as fiber reinforcement in addition to their primary function as a transport system, as reported for *Citrus maxima*. Moreover, a quasi-static mechanical analysis of different citrus peels would be promising to obtain information about the deformation of the peel at different thicknesses and gradations.

## Data Availability

The data is available upon reasonable request.
